# Comparative effectiveness of allopurinol versus febuxostat for preventing incident dementia in older adults: a propensity-matched analysis

**DOI:** 10.1186/s13075-018-1663-3

**Published:** 2018-08-03

**Authors:** Jasvinder A. Singh, John D. Cleveland

**Affiliations:** 1 Medicine Service, VA Medical Center, 510, 20th Street South, FOT 805B, Birmingham, AL 35233 USA; 2 Department of Medicine at School of Medicine, University of Alabama at Birmingham, 20th Street South, FOT 805B, Birmingham, AL 35294-0022 USA; 3 Division of Epidemiology at School of Public Health, University of Alabama at Birmingham, 1720 Second Avenue South, Birmingham, AL 35294-0022 USA; 4 University of Alabama, Faculty Office Tower 805B, 510 20th Street South, Birmingham, AL 35294-0022 USA

**Keywords:** Allopurinol, Febuxostat, Dementia, Older adults, Elderly, Medicare

## Abstract

**Background:**

The purpose of this study was to assess the comparative effectiveness of allopurinol versus febuxostat for preventing incident dementia in older adults.

**Methods:**

In a retrospective cohort study using Medicare claims data, we included patients newly treated with allopurinol or febuxostat (baseline period of 365 days without either medication). We used 5:1 propensity-matched Cox regression analyses to compare the hazard ratio (HR) of incident dementia with allopurinol versus febuxostat use and with allopurinol/febuxostat dose and duration.

**Results:**

Crude rates of incident dementia per 100,000 person-days were lower with higher daily dose: allopurinol less than 200, 200 to 299, and at least 300 mg/day with 12, 9, and 8 and febuxostat 40 and 80 mg/day with 9 and 8, respectively. In propensity-matched analyses, compared with allopurinol use, febuxostat use was not significantly different, and the HR of incident dementia was 0.79 (95% confidence interval (CI) 0.61, 1.03). Compared with allopurinol less than 200 mg/day, higher allopurinol doses (200 to 299 and at least 300 mg/day) and the febuxostat 40 mg/day dose were each associated with lower HRs of dementia: 0.80 (95% CI 0.64, 0.98), 0.59 (95% CI 0.50, 0.71), and 0.64 (95% CI 0.47, 0.86), respectively. Compared with allopurinol use for 1 to 180 days, longer allopurinol or febuxostat use durations were not significantly associated with differences in HR of dementia (range of 0.76 to 1.14).

**Conclusions:**

A dose-related reduction in the risk of dementia in older adults was noted with higher allopurinol dose and with febuxostat 40 mg daily dose. Future studies need to examine the mechanism of this benefit.

**Electronic supplementary material:**

The online version of this article (10.1186/s13075-018-1663-3) contains supplementary material, which is available to authorized users.

## Background

The association of uric acid levels and dementia is an emerging area of interest. An important unanswered question is whether urate-lowering therapy (ULT) use affects the risk of dementia. We recently showed that, compared with non-use, use allopurinol or febuxostat (the most commonly used ULTs) was not associated with any increase in the risk of dementia in older adults [1]. Important study limitations were that dose and duration were not examined and that allopurinol and febuxostat were not compared with each other. Allopurinol is a purine analog that non-selectively inhibits the xanthine oxido-reductase (XOR) system and other enzymes in purine and pyrimidine pathways, whereas febuxostat is a non-purine analog that selectively inhibits XOR [2]. XOR exists as xanthine oxidase (XO) or xanthine dehydrogenase (XDH) [3]. The XOR system catalyzes the two terminal reactions of purine metabolism in humans, the conversion of hypoxanthine into xanthine and xanthine into uric acid. The conversion of hypoxanthine into uric acid by XOR leads to the formation of superoxide species that increases oxidative stress. Additionally, uric acid has both a pro-oxidant action [4, 5] and an anti-oxidant action [6, 7] and is potentially neuroprotective based on its anti-oxidant effect. Thus, hyperuricemia may be associated with oxidative stress, which is implicated in the pathogenesis of dementia [8–10]. Allopurinol or febuxostat potentially reduces the risk of dementia by inhibiting XOR and reducing uric acid production; given its non-selective inhibition of other purine and pyrimidine pathways, allopurinol may differ in effect from febuxostat [2].

Dementia, characterized by progressive deterioration of cognitive ability and function, is a common disease of older adults and for the first time has replaced ischemic heart disease as the leading cause of death in England and Wales [11]. An estimated 36 million people worldwide had dementia in 2010, and the number is expected to double to 66 million by 2030 and quadruple to 115 million by 2050 [12]. Worldwide, the total estimated costs of dementia were USD $604 billion in 2010 [13] and were likely much higher recently [14]. Dementia is associated with the loss of independence and increased morbidity and mortality [15, 16]. Therefore, dementia is a significant public health problem of increasing impact.

A few studies showed that hyperuricemia was associated with a better cognition and a lower risk of dementia [17–19], whereas other studies showed an opposite effect [20–25]; that is, hyperuricemia was a risk factor for dementia. The 2016 European League Against Rheumatism (EULAR) treatment guideline for gout [26] cautioned against long-term lowering of serum urate of less than 3 mg/dL because of a potential risk of adverse neurologic outcome. This was based on low-quality evidence, mainly from studies in neurological conditions other than dementia. In contrast, a large French population-based study showed that hyperuricemia was associated with a higher risk of dementia in older adults [27]. Thus, robust studies are needed for a better understanding of this relationship. One way to examine the potential relationship or hyperuricemia with the risk of dementia, is to examine whether the use of ULT, or ULT dose or duration of use are associated with a reduced risk of dementia in older adults.

We hypothesized that in older adults (65 years or older; population at risk), ULT type, dose, and duration would be associated with the risk of dementia. Specifically, we assessed the hypotheses of whether (1) febuxostat is more effective than allopurinol in preventing dementia and (2) higher doses and longer duration of allopurinol or febuxostat use are associated with a greater risk reduction of dementia than lower doses or shorter use duration.

## Methods

### Study cohort

This is a retrospective cohort study of Medicare beneficiaries from 2006 to 2012. We examined the 5% random Medicare sample that contains all insurance claims for each beneficiary and has been widely used for epidemiological research. We obtained the data from the Centers for Medicare and Medicaid Services (CMS) Chronic Condition Data Warehouse. We abstracted data from the following files for each beneficiary: (1) a beneficiary summary file: demographics, including birthdate, death date, sex, and race, and monthly entitlement indicators (A/B/C/D); (2) part D file: prescription claims, dose, supply, and drug name; and (3) inpatient and outpatient claim files: diagnosis codes for each claim and claim dates. The Medicare beneficiaries were eligible for this study if they (1) were enrolled in Medicare fee-for-service with pharmacy coverage (parts A, B, and D) and not enrolled in a Medicare Advantage Plan, (2) resided in the US from 2006 to 2012, and (3) received new treatment with allopurinol or febuxostat, defined as any new filled prescription of allopurinol (or febuxostat) with a clean baseline period of 365 days without any allopurinol (or febuxostat) filled prescription (details in a section below). The institutional review board of the University of Alabama at Birmingham approved the study and waived the requirement for informed consent for this database study. Methods and results are being reported as recommended in the STROBE (Strengthening of Reporting in Observational studies in Epidemiology) statement.

### Exposure of interest: new treatment with allopurinol or febuxostat

A beneficiary began a new allopurinol (or febuxostat) treatment episode by filling an allopurinol prescription provided that they had not filled an allopurinol (or febuxostat) prescription in the previous 365 days. We assigned days of exposure for each allopurinol (or febuxostat) treatment episode, calculated on the basis of the days’ supply variable provided in the Medicare Part D file and included a 30-day residual period. Continuous allopurinol (or febuxostat) episode ended after 30 days of the end of allopurinol (or febuxostat) exposure. If there were more than 30 days between prescription fills, a new allopurinol (or febuxostat) exposure started. If a patient had prescriptions for both drugs, then they were considered exposed to the one that was prescribed second; for example, if a patient was taking allopurinol and got a new prescription of febuxostat, then he or she was considered to be on febuxostat only as of the febuxostat fill date.

If a patient received a 90-day supply of allopurinol (or febuxostat), then we considered them exposed for 120 days: 90 days of supply plus 30 days of residual period. We included a 30-day residual period to capture imperfect medication adherence and to account for any residual protective biological effects related to the medication itself. If the patient switched medications (allopurinol to febuxostat or vice versa), the 30-day latency did not apply and they were immediately classified as exposed to the new medication only. This was done since the ULTs achieve significant blood and tissue concentrations soon after initiation.

The main predictor of interest was febuxostat use, and allopurinol use was the reference category. We assessed all allopurinol doses (200 to 299 and at least 300 mg/day) and febuxostat dose (40 and 80 mg/day), and allopurinol less than 200 mg/day was the reference category. We calculated the daily allopurinol (or febuxostat) dose as the mean daily use for each continuous allopurinol (or febuxostat) episode. For each allopurinol (or febuxostat) treatment episode, we categorized the duration of use as 1 to 180 days, 181 to 365 days, and more than 1 year. Subjects contributed to the “none” category during periods in which they were not in an allopurinol or febuxostat treatment episode.

### Study outcome

The outcome of interest for our study was incident dementia, identified by the occurrence of a new diagnostic code for dementia with an absence of any prior diagnostic code in a 183-day baseline period before allopurinol or febuxostat initiation. We used the International Classification of Diseases, ninth revision, common modification (ICD-9-CM) code, 290.xx, 294.1x, or 331.2, for assessing dementia, as in the Quan-Charlson index [28], a validated and commonly used comorbidity index. These ICD-9-CM codes were shown to have high accuracy in the Medicare claims data [29], are valid for identifying patients with dementia with positive and negative predictive values of 96% and 98% (respectively) and specificity of 100% [30], and have been used in other studies to identify cohorts of dementia [31].

### Covariates

We assessed several important covariates, including patient demographics, medical comorbidity, and the use of medications for cardiovascular diseases for the baseline period for each episode, obtained from the Medicare denominator and other claims files. Biological variables such as age, sex, and race/ethnicity were included, as were cardiovascular medications and the Charlson-Romano comorbidity index score, a validated comorbidity index developed for claims data analysis [32]. The Charlson-Romano comorbidity index is an adaptation of the Charlson index, the most commonly used comorbidity index in research studies. It is a weighted index that includes comorbidities such as diabetes, myocardial infarction, congestive heart failure, cerebrovascular disease, liver disease, pulmonary disease, peripheral vascular disease, and rheumatic disease. We included statins, beta-blockers, diuretics, and angiotensin-converting enzyme (ACE) inhibitors since these cardiovascular medications (as markers of active cardiovascular disease or by an independent effect), coronary artery disease (CAD), and risk factors for CAD (tobacco use disorder, hyperlipidemia, and hypertension) might impact the risk of dementia.

### Statistical analyses

We compared baseline characteristics of episodes with versus without incident dementia and calculated crude incidence rates of incident dementia for new allopurinol (or febuxostat) episodes. For comparative efficacy of allopurinol versus febuxostat, we performed propensity score–matched (matched 5:1 on propensity score) Cox proportional hazard regression analyses to control for differences between patients exposed to allopurinol versus febuxostat. Propensity matching included age, sex, race, Charlson-Romano comorbidity score, region, each Charlson-Romano comorbidity, risk factors for CAD, and the use of medications for cardiovascular diseases (statins, beta-blockers, diuretics, and ACE inhibitors). The analyses for allopurinol and febuxostat daily dose used allopurinol less than 200 mg daily dose as the reference category, and the analyses for allopurinol and febuxostat use duration considered allopurinol use of 1 to 180 days as the reference category.

We used multivariable-adjusted Cox proportional hazard regression models and Huber-White “Sandwich” variance estimator to account for correlations between observations from the same patient [33] and calculated the hazard ratio (HR) of incident dementia for febuxostat use versus allopurinol use (reference category). We conducted additional sensitivity analyses to test the robustness of findings by repeating the propensity-matched analyses only in patients with gout.

## Results

### Patient characteristics and crude rates in patients receiving allopurinol or febuxostat

**Fig. 1:**
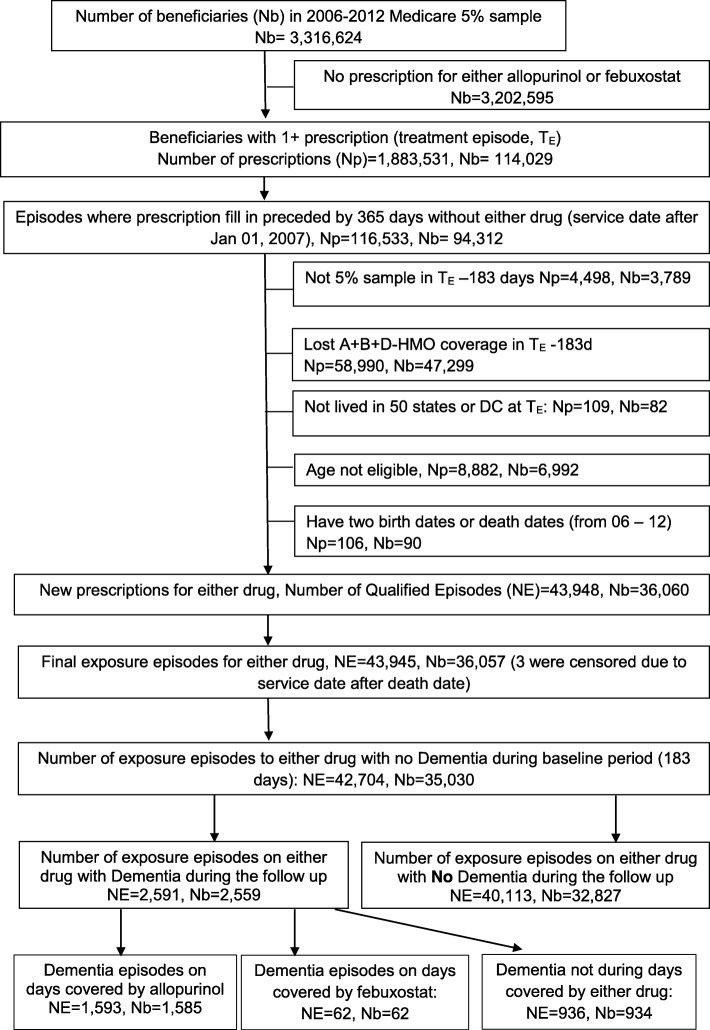
Patient selection flow chart. The flow chart shows the selection of new allopurinol exposure episodes after applying all the eligibility criteria, including the absence of any allopurinol or febuxostat filled prescription in the baseline period of 365 days (new user design) and an absence of dementia. We found 42,704 new allopurinol or febuxostat exposure episodes in 35,030 patients. Of these, 2591 ended in incident dementia and 40,113 ended without incident dementia. *We followed each eligible patient with a new filled allopurinol or febuxostat prescription until the patient lost full Medicare coverage, had dementia (the outcome of interest), died, or reached the each of the study period on December 31, 2012, whichever came first. For some of these patients, dementia occurred on days covered by allopurinol exposure (*n* = 1593) or febuxostat exposure (*n* = 62), yet other patients had periods of no allopurinol or febuxostat use after an initial qualifying prescription during which dementia occurred (*n* = 936 exposure episodes). Abbreviations: *Nb* number of beneficiaries; *NE* number of qualified episodes of new allopurinol or febuxostat prescriptions, *Np* number of allopurinol or febuxostat prescriptions; *T*_*E*_ treatment episodes

We found 42,704 new allopurinol or febuxostat treatment episodes in 35,030 patients, and 2591 of these episodes ended in incident dementia (Fig. 1). Compared with patients with no dementia, patients with a new diagnosis of dementia were older, more likely to be female, White, and living in the South and had a higher Charlson-Romano comorbidity index (Table 1). The crude incidence of dementia in people with allopurinol use, or febuxostat use was 10 and 9 per 100,000 person-days, respectively (Table 2). The crude incidence of dementia by daily ULT dose was as follows: allopurinol less than 200 mg, 200 to 299 mg, and at least 300 mg daily: 12, 9, and 8 per 100,000 person-days; and febuxostat 40 and 80 mg daily, 9 and 8 per 100,000 person-days (Table 2). The mean study follow-up time was 922.5 days, and 86.1% of the people receiving the allopurinol or febuxostat prescription (36,760 out of 42,704) had gout.

**Table 1:** Demographic and clinical characteristics of all new episodes* of allopurinol or febuxostat use

	All episodes	Incident dementia* during the follow-up		*P* value
		Yes	No	
Total, N (episodes)	42,704	2591	40,113	
Age in years, mean (SD)	76.0 (7.38)	80.8 (7.36)	75.7 (7.27)	< 0.0001
Sex, N (%)				< 0.0001
Male	22,125 (51.8%)	1099 (42.4%)	21,026 (52.4%)	
Female	20,579 (48.2%)	1492 (57.6%)	19,087 (47.6%)	
Race/Ethnicity, N (%)				< 0.0001
White	33,409 (78.2%)	1975 (76.2%)	31,434 (78.4%)	
Black	5317 (12.5%)	391 (15.1%)	4926 (12.3%)	
Hispanic	898 (2.1%)	65 (2.5%)	833 (2.1%)	
Asian	2073 (4.9%)	118 (4.6%)	1955 (4.9%)	
Native American	129 (0.3%)	10 (0.4%)	119 (0.3%)	
Other/unknown	878 (1.3%)	32 (1.2%)	846 (2.1%)	
Region, N (%)				< 0.0001
Midwest	10,488 (24.6%)	582 (22.5%)	9906 (24.7%)	
Northeast	6901 (16.2%)	526 (20.3%)	6375 (15.9%)	
South	17,351 (40.6%)	1081 (41.7%)	16,270 (40.6%)	
West	7964 (18.6%)	402 (15.5%)	7562 (18.9%)	
Charlson-Romano comorbidity score, mean (SD)	1.77 (2.09)	2.31 (2.26)	1.74 (2.08)	< 0.0001

Abbreviation: *SD* standard deviation

*Baseline period of 365 days without allopurinol or febuxostat use and without dementia

**Table 2:** Crude incidence rate of dementia with allopurinol versus febuxostat exposure

	Person-days of follow-up	Number of cases of incident dementia	Dementia incidence rate per 100,000 person-days
Allopurinol	16,999,091	1593	10
Febuxostat	774,291	62	9
Allopurinol duration			
1–180 days	7,775,101	834	11
181–365 days	3,311,650	286	9
>1 year	5,912,340	473	9
Febuxostat duration			
1–180 days	435,872	35	9
181–365 days	167,335	14	9
>1 year	171,084	13	8
Allopurinol dose			
< 200 mg/day	7,701,139	899	12
200–299 mg/day	3,091,767	251	9
≥300 mg/day	6,206,185	443	8
Febuxostat dose			
40 mg/day	634,925	51	9
80 mg/day	139,366	11	8

*Drug exposure considered up to 30 days after last day of medication fill/refill (except when switched to the other ULT, i.e., switched from allopurinol to febuxostat or vice versa); baseline period was 365 days, that is, each new exposure was defined as no previous exposure (allopurinol or febuxostat) in the baseline period

### Propensity-matched analysis of allopurinol or febuxostat use: risk of dementia

Most of the significant differences noted between allopurinol and febuxostat users before propensity matching (Additional file 1) were eliminated and reduced to non-significant differences in 5:1 propensity-matched cohorts (Additional file 1). In propensity-matched analyses with 12,135 episodes of exposure to allopurinol and 2427 episodes of febuxostat exposure (5:1 matching), compared with allopurinol use (all doses), febuxostat use (all doses) was not significantly different regarding the risk of incident dementia, and the HR was 0.79 (95% confidence interval (CI) 0.61, 1.03) (Table 3). Compared with allopurinol less than 200 mg/day, higher allopurinol doses (200 to 299 mg/day and at least 300 mg/day) and febuxostat 40 mg/day dose were each associated with significantly lower HRs of dementia: 0.80 (95% CI 0.64, 0.98), 0.59 (95% CI 0.50, 0.71), and 0.64 (95% CI 0.47, 0.86), respectively (Table 3); febuxostat 80 mg/day was not significantly different, and the HR was 0.66 (95% CI 0.36, 1.19). Compared with allopurinol use of 1 to 180 days, longer febuxostat or allopurinol use durations were not significantly associated with differences in HR of dementia, which ranged from 0.76 to 1.14 (Table 3). Sensitivity analyses limited to patients with gout confirmed all the findings above, and there was minimal attenuation of HRs and no change in statistical significance (Additional file 2).

**Table 3:** Propensity-score adjusted association of allopurinol or febuxostat with the hazard of incident dementia in patients who received allopurinol or febuxostat with a clean baseline period of 365 days*

	Hazard ratio (95% CI)	*P* value
Allopurinol	Ref	
Febuxostat	0.79 (0.61, 1.03)	0.09
Urate-lowering therapy (ULT) dose		
Allopurinol <200 mg/day	Ref	
Allopurinol 200–299 mg/day	**0.80 (0.64, 0.98)**	**0.03**
Allopurinol ≥300 mg/day	**0.59 (0.50, 0.71)**	**<0.0001**
Febuxostat 40 mg/day	**0.64 (0.47, 0.86)**	**0.003**
Febuxostat 80 mg/day	0.66 (0.36, 1.19)	0.17
ULT duration		
Allopurinol, 1–180 days	Ref	
Allopurinol, 181–365 days	1.14 (0.86, 1.53)	0.36
Allopurinol, >1 year	1.12 (0.84, 1.49)	0.46
Febuxostat, 1–180 days	0.76 (0.53, 1.08)	0.13
Febuxostat, 181–365 days	1.09 (0.61, 1.94)	0.77
Febuxostat, >1 year	0.82 (0.44, 1.53)	0.54

Abbreviations: *CI* confidence interval, *Ref* referent category.

*Baseline period of 365 days without allopurinol or febuxostat use and without any diagnosis of dementia; A bold font indicates associations that are statistically significant with a p-value <0.05

## Discussion

In this study of Americans who were at least 65 years old, higher doses of allopurinol and febuxostat 40 mg/day, compared with low-dose allopurinol (<200 mg/day), were associated with a lower hazard of incident dementia. Compared with allopurinol use duration of 1 to 180 days, longer durations of allopurinol or febuxostat use were not associated with significant reductions of hazard of dementia. Overall, febuxostat did not differ from allopurinol in reducing the risk of dementia. All of our findings were reproduced in propensity-matched analyses limited to people with gout, and there was minimal attenuation of HRs and no change in significance. Since we were inherently interested in examining comparative effectiveness of allopurinol versus febuxostat in people with gout, we did not include gout in propensity matching. Nevertheless, sensitivity analyses performed in people with gout to address this issue essentially reproduced the main findings, as expected. These findings indirectly support and extend the findings of studies that have shown that hyperuricemia is associated with a higher risk of dementia [20–25], including a recent large population-based study [27], although some studies do not agree [17–19]. The existing controversy related to hyperuricemia and the risk of dementia [34] and the lack of studies of ULT led us to assess the comparative efficacy of allopurinol and febuxostat for preventing dementia. Several findings from our study merit further discussion.

Only one previous Taiwanese study reported that, compared with patients without gout, patients with gout receiving ULT had much lower odds of dementia, 0.71 (95% CI 0.65, 0.78), while untreated gout patients did not differ, 0.97 (95% CI 0.87, 1.09) [17]. This indicated a potential beneficial effect related to ULT use, but the evidence was indirect at best. We found that, compared with allopurinol less than 200 mg/day, allopurinol 200 to 299 and at least 300 mg/day were associated with 20% and 41% reductions (respectively) in the hazard of dementia. High-dose allopurinol reduces vascular oxidative stress significantly more than the conventional doses [35, 36], which supports this finding. Allopurinol inactivates XOR and, at high concentrations, can scavenge hydroxyl radicals [37]. Febuxostat, the new XOR inhibitor, also has oxidative stress reduction properties [38, 39]. Evidence has linked oxidative stress and mitochondrial dysfunction [8–10] to neurodegenerative processes in dementia. Oxidative damage to mitochondrial DNA demonstrates an age-dependent increase in human brain [40]. Animal studies also showed that associated oxidative damage in brain [41] and upregulation of genes relating to mitochondrial metabolism and apoptosis in neurons [42] precede Aβ-amyloid deposition and neuronal injury. Thus, inhibition of oxidative stress with febuxostat and higher doses of allopurinol might explain the associated reduced risk of dementia, compared with lower allopurinol doses. It is possible that other indirect urate-related mechanisms (for example, decreased cardiovascular burden) underlie the neuroprotective effect observed with higher doses of ULT in this study.

We noted a biologic gradient with increasing allopurinol dose that satisfied one of Bradford-Hill’s criteria for causation versus association [43]. Interestingly, of the three studies that found gout to be protective of dementia, two did not adjust for ULT use [18, 19]. One study that separated patients with gout by treatment found that, compared with matched controls, only patients with ULT-treated gout had a lower hazard of dementia (HR 0.69, 95% CI 0.64, 0.75) but not untreated patients (HR 0.963, 95% CI 0.84, 1.03) [17]. These findings again indicated to us that ULT use might explain these associations of hyperuricemia to lower risk of dementia, which were contrary to other studies that found hyperuricemia to be a risk factor for dementia.

Dementia is a slow process, and a relatively short treatment duration might not be sufficient to counteract the detrimental effect of many years of hyperuricemia with regard to the increased risk of dementia. This may lead to underestimations of the neuroprotective effect of ULT and also may explain the lack of effect of ULT duration on the risk of dementia. Studies of longer duration are needed to further address the questions related to the duration of ULT use and potential reduction of the risk of dementia.

Our finding related to allopurinol dose is interesting and has important clinical implications. Allopurinol, ranging from 100 to 300 mg/day with a mean dose of 230 mg/day, is commonly used in suboptimal doses in patients with gout [44]; less than 50% of those treated at these allopurinol doses achieve the target serum urate of less than 6 mg/dL [45], an important goal associated with lower flare rate [46]. Therefore, higher allopurinol doses are needed in a significant proportion of patients with gout for an appropriate treatment of hyperuricemia and can be given safely [47]. Evidence from studies such as this and others [48] should provide a strong motivation for clinicians to use appropriate (that is, higher) doses of allopurinol to achieve maximum benefits for patients. Additional non-arthritic benefits, such as these, can be considered in decision-making regarding ULT and its dose.

Our study failed to confirm our hypothesis that febuxostat is significantly more effective than allopurinol for the reduction of risk of dementia, which was based on previously observed greater oxidative stress reduction with febuxostat compared with allopurinol [49]. This may be due to either a true lack of difference in mechanism of action between allopurinol and febuxostat related to dementia risk or few events in those exposed to febuxostat. In our study, the majority of febuxostat use was 40 mg/day (82%), while the allopurinol use was split between doses: less than 200 mg/day (45%), 200 to 299 mg/day (18%), and at least 300 mg/day (37%). The lowest marketed dose of febuxostat (40 mg/day) is as efficacious as the allopurinol 300 mg/day dose [50]. Thus, non-significant differences between allopurinol and febuxostat can be explained by the differences in commonly used doses of allopurinol versus febuxostat.

Our study has several limitations that must be considered while interpreting the findings. We examined only older Americans, who were Medicare beneficiaries. Therefore, these findings can be generalized only to Americans 65 years or older; however, this patient population is at high risk of dementia. We examined the most commonly used ULTs: allopurinol and febuxostat. Because few patients received doses higher than 300 mg/day (real-world practice), we were unable to examine the association with higher allopurinol doses (that is, 600 mg/day). We conducted propensity-matched analyses to avoid channeling bias, and most variables matched very closely in propensity-matched cohorts. We included several potential confounders to reduce the risk of confounding bias; however, residual confounding is still possible. Misclassification bias is possible for dementia; however, we used validated codes for dementia [31] shown to have high accuracy [30]. These codes have been used in studies using Medicare claims data [29] and are used in the Quan-Charlson index [28], a very commonly used comorbidity index in research studies. Sensitivity analyses performed by limiting ourselves to one specific code for dementia (290.xx), as in the Charlson-Deyo index, confirmed the findings in the main analysis. Our study was not designed to answer the following questions, which need to be answered by future studies: (1) can allopurinol or febuxostat reduce the progression of cognitive decline in patients with early dementia, (2) does the comparative effectiveness of allopurinol versus febuxostat differ by the type of dementia (that is, Alzheimer’s versus vascular dementia), and (3) does the extent of lowering of serum urate correlate with the prevention of dementia and differ for the types of dementia?

Our study has several strengths. We used a new (allopurinol/febuxostat) user design and required all patients to be free of dementia at baseline for a rigorous study design. We examined a representative national sample of the older adults in the US, adjusted for important confounders and covariates, and conducted sensitivity analyses to make sure our results were robust.

## Conclusions

We found that, compared with lower allopurinol dose (<200 mg/day), higher allopurinol doses and febuxostat 40 mg/day were associated with a lower risk of a new diagnosis of dementia. This association does not imply causation. This finding provides a rationale for the use of therapeutic allopurinol doses for treatment of conditions such as gout, with an added potential benefit of lower risk of dementia. Mechanistic studies are now needed to better understand how and why this ULT dose-related benefit of reduction of risk of dementia occurs.

## Additional files

Additional file 1: Comparison of all variable used in propensity match before and after 5:1 matching. This file shows the differences in important patient characteristics between allopurinol and febuxostat users before propensity matching. As is evident, several variables differed significantly. The last three columns show the distribution of important variables in the propensity-matched allopurinol use and febuxostat use cohorts. The table shows that all variables matched well. (DOCX 27 kb)

Additional file 2: Sensitivity analyses for propensity-score adjusted association of allopurinol or febuxostat with hazard of incident dementia (limited to patients with gout). This table shows sensitivity analyses with propensity-score adjusted analyses limited to patients with gout. Main results were replicated in this sensitivity analyses without any attenuation of estimates for dose and duration effects. (DOCX 15 kb)

## Abbreviations

ACE: Angiotensin-converting enzyme
CAD: Coronary artery disease
CI: Confidence interval
HR: Hazard ratio
ICD-9-CM: International Classification of Diseases, ninth revision, common modification
ULT: Urate-lowering therapy
XOR: Xanthine oxido-reductase system
