# Supplementary material for: Comparative effectiveness of allopurinol versus febuxostat for preventing incident dementia in older adults: a propensity-matched analysis
Source: Arthritis Res Ther. 2018 Aug 3;20:167. doi: 10.1186/s13075-018-1663-3 (PMC6091090; doi:10.1186/s13075-018-1663-3)
Supplement: Supplementary file 1 — Comparison of all variable used in propensity match before and after 5:1 matching. This file shows the differences in important patient characteristics between allopurinol and febuxostat users before propensity matching. As is evident, several variables differed significantly. The last three columns show the distribution of important variables in the propensity-matched allopurinol use and febuxostat use cohorts. The table shows that all variables matched well. (DOCX 27 kb) [file 13075_2018_1663_MOESM1_ESM.docx]

**Additional File 1.** Comparison of all variables used in propensity match showing before and after matching

|  | **Pre-match** | | | **Post-match** | | |  |  |
| --- | --- | --- | --- | --- | --- | --- | --- | --- |
|  | Allopurinol* | Febuxostat* | p-value** | Allopurinol* | Febuxostat* | p-value** |  |  |
| **Total episodes** | **40,090** | **2,614** |  | **12,135** | **2,427** |  |  |  |
| Age category |  |  |  |  |  |  |  |  |
| 65-75 | 18992 (47.4%) | 1206 (46.1%) | Ref | 5679 (46.8%) | 1140 (47.0%) | 0.84 |  |  |
| 75-85 | 15170 (37.8%) | 1007 (38.5%) | 0.57 | 4577 (37.7%) | 929 (38.3%) | 0.48 |  |  |
| >85 | 5928 (14.8%) | 401 (15.3%) | 0.32 | 1879 (15.5%) | 358 (14.8%) | 0.14 |  |  |
| Gender |  |  |  |  |  |  |  |  |
| Male | 20771 (51.8%) | 1354 (51.8%) | Ref | 5917 (48.8%) | 1171 (48.3%) | 0.57 |  |  |
| Female | 19319 (48.2%) | 1206 (48.2%) | 0.41 | 6218 (51.2%) | 1256 (51.8%) | 0.58 |  |  |
| Race |  |  |  |  |  |  |  |  |
| White | 31412 (78.4%) | 1997 (76.4%) | Ref | 9478 (78.1%) | 1860 (76.6%) | 0.19 |  |  |
| Black | 5042 (12.6%) | 275 (10.5%) | **0.035** | 1212 (10.0%) | 258 (10.6%) | 0.12 |  |  |
| Other | 3636 (9.1%) | 342 (13.1%) | **0.0002** | 1445 (11.9%) | 309 (12.7%) | 0.07 |  |  |
| Charlson Romano score mean (SD) | 1.76 (2.09) | 2.05 (2.17) | **<0.0001** | 1.87 (2.10) | 1.95 (2.10) | **<0.0001** |  |  |
| Beta-blockers | 3525 (8.8%) | 217 (8.3%) | 0.73 | 893 (7.4%) | 186 (7.7%) | 0.39 |  |  |
| ACE Inhibitors | 2586 (6.5%) | 91 (3.5%) | **<0.0001** | 344 (2.8%) | 78 (3.2%) | 0.09 |  |  |
| Diuretics | 4062 (10.1%) | 250 (9.6%) | 0.26 | 1063 (8.8%) | 222 (9.2%) | 0.31 |  |  |
| Statins | 3285 (8.2%) | 191 (7.3%) | 0.09 | 788 (6.5%) | 171 (7.1%) | 0.10 |  |  |
| Charlson Romano Comorbidities |  |  |  |  |  |  |  |  |
| Myocardial Infarction | 1160 (2.9%) | 119 (4.6%) | 0.52 | 451 (3.7%) | 101 (4.2%) | 0.08 |  |  |
| Diabetes | 14865 (37.1%) | 995 (38.1%) | **<0.0001** | 4370 (36.0%) | 909 (37.5%) | 0.06 |  |  |
| Peripheral vascular disease | 4725 (11.8%) | 343 (13.1%) | **0.0002** | 1411 (11.6%) | 301 (12.4%) | 0.08 |  |  |
| Cerebrovascular disease | 3356 (8.4%) | 200 (7.7%) | **<0.0001** | 837 (6.9%) | 177 (7.3%) | 0.25 |  |  |
| Congestive heart failure | 8643 (21.6%) | 608 (23.3%) | **<0.0001** | 2624 (21.6%) | 536 (22.1%) | 0.44 |  |  |
| Chronic pulmonary disease | 6271 (15.6%) | 515 (19.7%) | **<0.0001** | 2240 (18.5%) | 459 (18.9%) | 0.41 |  |  |
| Connective tissue disease | 1161 (2.9%) | 131 (5.0%) | **<0.0001** | 559 (4.6%) | 115 (4.7%) | 0.63 |  |  |
| Peptic ulcer disease | 473 (1.2%) | 50 (1.9%) | **0.016** | 185 (1.5%) | 36 (1.5%) | 0.79 |  |  |
| Mild liver disease | 287 (0.7%) | 26 (1.0%) | **0.015** | 112 (0.9%) | 21 (0.9%) | 0.63 |  |  |
| Hemiplegia or paraplegia | 162 (0.4%) | 18 (0.7%) | 0.99 | 72 (0.6%) | 14 (0.6%) | 0.87 |  |  |
| Malignancy | 5729 (14.3%) | 255 (9.8%) | **<0.0001** | 1065 (8.8%) | 239 (9.9%) | **0.006** |  |  |
| Moderate or severe liver disease | 72 (0.2%) | 3 (0.1%) | **<0.0001** | 14 (0.1%) | 3 (0.1%) | 0.85 |  |  |
| Metastatic solid tumor | 429 (1.1%) | 11 (0.4%) | **<0.0001** | 53 (0.4%) | 11 (0.5%) | 0.85 |  |  |
| AIDS/HIV | 26 (0.1%) | 3 (0.1%) | 0.16 | 13 (0.1%) | 3 (0.1%) | 0.71 |  |  |
| Moderate or severe renal disease | 8291 (20.7%) | 175 (6.7%) | **<0.0001** | 880 (7.3%) | 167 (6.9%) | 0.28 |  |  |
| Diabetes with end organ damage | 3697 (9.2%) | 368 (14.1%) | 0.11 | 1450 (12.0%) | 316 (13.0%) | **0.02** |  |  |
| Tobacco use disorder | 794 (2.0%) | 46 (1.8%) | 0.35 | 204 (1.7%) | 42 (1.7%) | 0.77 |  |  |
| Hypertension | 29896 (74.6%) | 2142 (81.9%) | **<0.0001** | 9944 (81.9%) | 1982 (81.7%) | 0.81 |  |  |
| Hyperlipidemia | 19517 (48.7%) | 1423 (54.4%) | **0.001** | 6502 (53.6%) | 1302 (53.7%) | 0.94 |  |  |
| Coronary artery disease | 12744 (31.8%) | 894 (34.2%) | 0.77 | 3947 (32.5%) | 808 (33.3%) | 0.30 |  |  |
| Region |  |  |  |  |  |  |  |  |
| Midwest | 10025 (25.0%) | 483 (18.5%) | **<0.0001** | 2193 (18.1%) | 457 (18.8%) | 0.17 |  |  |
| Northeast | 6451 (16.1%) | 473 (18.1%) | 0.19 | 2119 (17.5%) | 423 (17.4%) | 0.95 |  |  |
| South | 16262 (40.6%) | 1063 (40.7%) | **0.011** | 5184 (42.7%) | 1005 (41.4%) | 0.12 |  |  |
| West | 7352 (18.3%) | 595 (22.8%) | Ref | 2639 (21.8%) | 542 (22.3%) | 0.33 |  |  |
| Propensity score | -- | -- | -- | 0.0849 (0.04595) | 0.0853 (0.04618) |  |  |  |
| *All numbers are N (%), unless otherwise specified  **p-values are from logistic regression performed on all variables  SD, standard deviation | | | | | | |  |  |
