# Supplementary material for: Comparative effectiveness of allopurinol versus febuxostat for preventing incident dementia in older adults: a propensity-matched analysis
Source: Arthritis Res Ther. 2018 Aug 3;20:167. doi: 10.1186/s13075-018-1663-3 (PMC6091090; doi:10.1186/s13075-018-1663-3)
Supplement: Supplementary file 2 — Sensitivity analyses for propensity-score adjusted association of allopurinol or febuxostat with hazard of incident dementia (limited to patients with gout). This table shows sensitivity analyses with propensity-score adjusted analyses limited to patients with gout. Main results were replicated in this sensitivity analyses without any attenuation of estimates for dose and duration effects. (DOCX 15 kb) [file 13075_2018_1663_MOESM2_ESM.docx]

**Additional File 2.** Sensitivity analyses for propensity-score adjusted association of allopurinol or febuxostat with hazard of incident dementia, limited to patients with gout

|  | HR (95% CI) | P-value |
| --- | --- | --- |
| Allopurinol | Ref |  |
| Febuxostat | 0.84 (0.64, 1.11) | 0.22 |
|  |  |  |
| Dose |  |  |
| Allopurinol <200 mg/day | Ref |  |
| Allopurinol 200-299 mg/day | **0.77 (0.62, 0.96)** | **0.021** |
| Allopurinol ≥300 mg/day | **0.65 (0.54, 0.78)** | **<0.0001** |
| Febuxostat 40 mg/day | **0.70 (0.52, 0.95)** | **0.023** |
| Febuxostat 80 mg/day | 0.67 (0.36, 1.27) | 0.22 |
|  |  |  |
| Duration |  |  |
| 1-180 days \| allopurinol | Ref |  |
| 181-365 days \| allopurinol | 1.17 (0.87, 1.57) | 0.30 |
| >1 year \| allopurinol | 1.03 (0.78, 1.38) | 0.82 |
| 1-180 days \| febuxostat | 0.79 (0.54, 1.14) | 0.21 |
| 181-365 days \| febuxostat | 1.20 (0.67, 2.14) | 0.54 |
| >1 year \| febuxostat | 0.85 (0.46, 1.58) | 0.61 |
| HR, Hazard ratio; CI, confidence interval; Ref, referent category | | |
